# VR4Health: Personalized teaching and learning anatomy using VR

**DOI:** 10.1007/s10916-020-01550-5

**Published:** 2020-03-19

**Authors:** M. Fairén, J. Moyés, E. Insa

**Affiliations:** 1 ViRVIG Group, Universitat Politècnica de Catalunya, Barcelona, Spain; 2 GIEES Group, Campus Docent Sant Joan de Déu-Fundació privada, Barcelona, Spain

**Keywords:** Virtual reality, Self-learning, Health sciences, Nursing

## Abstract

Virtual Reality (VR) is being integrated into many different areas of our lives, from industrial engineering to video-games, and also including teaching and education. We have several examples where VR has been used to engage students and facilitate their 3D spatial understanding, but can VR help also teachers? What are the benefits teachers can obtain on using VR applications? In this paper we present an application (VR4Health) designed to allow students to directly inspect 3D models of several human organs by using Virtual Reality systems. The application is designed to be used in an HMD device autonomously as a self-learning tool and also reports information to teachers in order that he/she becomes aware of what the students do and can redirect his/her work to the concrete necessities of the student. We evaluate both the students’ and the teachers’ perception by doing an experiment and asking them to fill-in a questionnaire at the end of the experiment.

## Introduction

Teaching human anatomy by using only 2D images has been always complicated. Advanced technologies have been the clue to clarify all the 3D anatomy structures by using directly 3D models to interact with (www.visiblebody.com; www.anatomylearning.com). This is even more useful when the student can use Virtual Reality (VR) systems to explore and see what the teacher has explained in class.

Following the good experience we had with an anatomy teaching activity carried out with nursing students in Campus Docent Sant Joan de Déu [5], we now present a new application, VR4Health, to be a self-learning tool that allows the teacher to monitor and guide the learning process of the student.

Self-learning is the way of learning that is equipping people with relevant skills for their daily activities. Although this requires lots of discipline, it has some proven advantages:

- Developing problem solving skills
- Stress-free learning process
- Learning experience becomes more meaningful
- Learning is led by curiosity
- Choose your mode of learning

By allowing VR4Health to be also a teaching facilitator tool we want to avoid some problems when student is completely alone in self-learning:

- The complete freedom of the student to learn, because this requires much discipline and auto-control.
- The missing guidance and help from teacher to student, that is required by many students (see results in Section “Evaluation and results”).

The paper is structured as follows: Section “Related work” reviews some related work, Section “VR4Health, the project” explains the VR4Health project, Section “Experiment design” describes the experiment for the user study whose results are exposed in Section “Evaluation and results”, and finally we conclude in Section “Conclusions”.

## Related work

Virtual Reality has been present in the educational field for more than half a century but its adoption is not yet extensive. The use of virtual reality learning environments (VRLEs) (see [1, 4, 15]) and the technologies related to them has grown in recent years due to their benefits compared to classical education, the educational and education opportunities they offer [17] and the ability to change the social dynamics of learning environments through transformed social interaction (see [2]).

Thus, we see the use of VR in different areas of the educational field, being the medical one of the most developed. We can emphasize: a) training of medical or surgical procedures [3, 13, 14, 16, 18, 21], and b) learning and understanding of anatomomorphological aspects of difficult visualization or access [6, 22]. In both cases, the aim is to improve professional competence and ultimately reduce human errors in professional practice.

Nevertheless, technology costs and logistics constitute some of the limitations of their use (see [11]) that teachers and education institutions have to deal with. In this sense, what is interesting is to ask what do teachers expect to win with the use of VR in their classes and what are their motivations [19].

One of the reasons why most teachers use VR, as specified by Kavanagh et al. [11], is to increase the motivation of students. In this case, they justify their use, referring to factors such as constructivist pedagogy, collaboration and gamification in the design of experiences. Keskitalo [12] focuses the attention on the pedagogical use of VR in education and training based on simulation. He shows that teachers have different conceptions about learning. They find that pedagogical models are not consistent or not well defined. Hence the concern to specify and develop a pedagogical model for the use of VR.

The effectiveness of VR in learning is another topic of debate among teachers. Some experiences of the usage of VR for the learning of human anatomy and physiology have shown that students consider VR as a useful tool and that it can facilitate their study and comprehension. Fairén et al. [5] and Yildrim [23] coincide in considering experience and interaction as key elements on the learning process. Games, for instance, are seen to be preferred in front of images or video, due to the sense of reality that they offer and the capacity to interact with the environment.

In self-learning the centrality of learning lies with the individual (see [9]). In it, students should be independent and learn what they are supposed to learn meanwhile teachers has to guide them and provide them with abilities and support so they can become self-directed and become responsible adults for acquire competencies [10]. So self-learning, supporting lifelong learning, is different from the traditional class which is guided and dominated by the curriculum and the evaluation tests. In this sense, the evaluation of self-learning activities need to be different from those traditional. Alternatives to conventional examinations are required and they need allow to evaluate the motivation, the level of interest and the participation in communities of learning combined with techniques for the longitudinal long term evaluation (see [7]).

Applied to nursing students, schools and universities must have the right tools to measure the self-directed learning (SDL) abilities. Cheng, Kuo, Lin and Lee-Hsieh [20], for example, developed an instrument to measure the SDL abilities of nursing students and test the validity and reliability of the instrument. On the other hand, technological development can be exploited to generate tools that promote lifelong learning while attaining specific learning objectives. Applied to the field of VR, the development of technology and VR applications is presented as an opportunity to cover both objectives.

An example of self-learning VR application is the Human Anatomy VR tool from Virtual Medicine (http:www.medicinevirtual.com) which allows the student to visualize and interact in 3D with a set of human anatomical structures and also offers the student the possibility of doing a self-evaluation by using quizzes. This tool does not involve the teacher in the learning and therefore does not allow tracking of student process. It is difficult to monitor the student discipline and recover the motivation when is lost.

The tool presented in this paper is also usable as a self-learning tool by allowing the visualization and interaction in 3D with a set of human anatomical structures but without including quizzes to do a self-evaluation. Our application is thought as a training tool where the teacher is able to follow the learning process and to give feedback to the student. Our tool allows the teacher to obtain data on motivation, level of interest and possible problems, or at least, promotes that the teacher formulates these questions later to the student at the time of feedback.

## VR4Health, the project

VR4Health was inspired by the anatomy teaching activity carried out with nursing students from Campus Docent Sant Joan de Déu [5]. Students attended to a seminar in the Barcelona VR Center (UPC) where they may experiment with 3D models of several anatomical organs by using two different VR systems, a powerwall and a CAVE. Each VR session is given to a small group of 15-20 students divided in two subgroups and directed by two assistant teachers who adopt the role of learning facilitator while the students are interacting with the models. This activity is being done every course since 2014 and is really appreciated by students and teachers.

Following the good reception of this activity and taking into account that the new HMD devices appeared in recent years have an affordable price for the consumer, we decided to adapt the application to be used in an HMD device and we extended it to be used autonomously as a self-learning tool. VR4Health (the new application) also includes several facilitator mechanisms in order that the teacher can modify and adapt the teaching activities to the student necessities.

### The self-learning tool

**Fig. 1:**
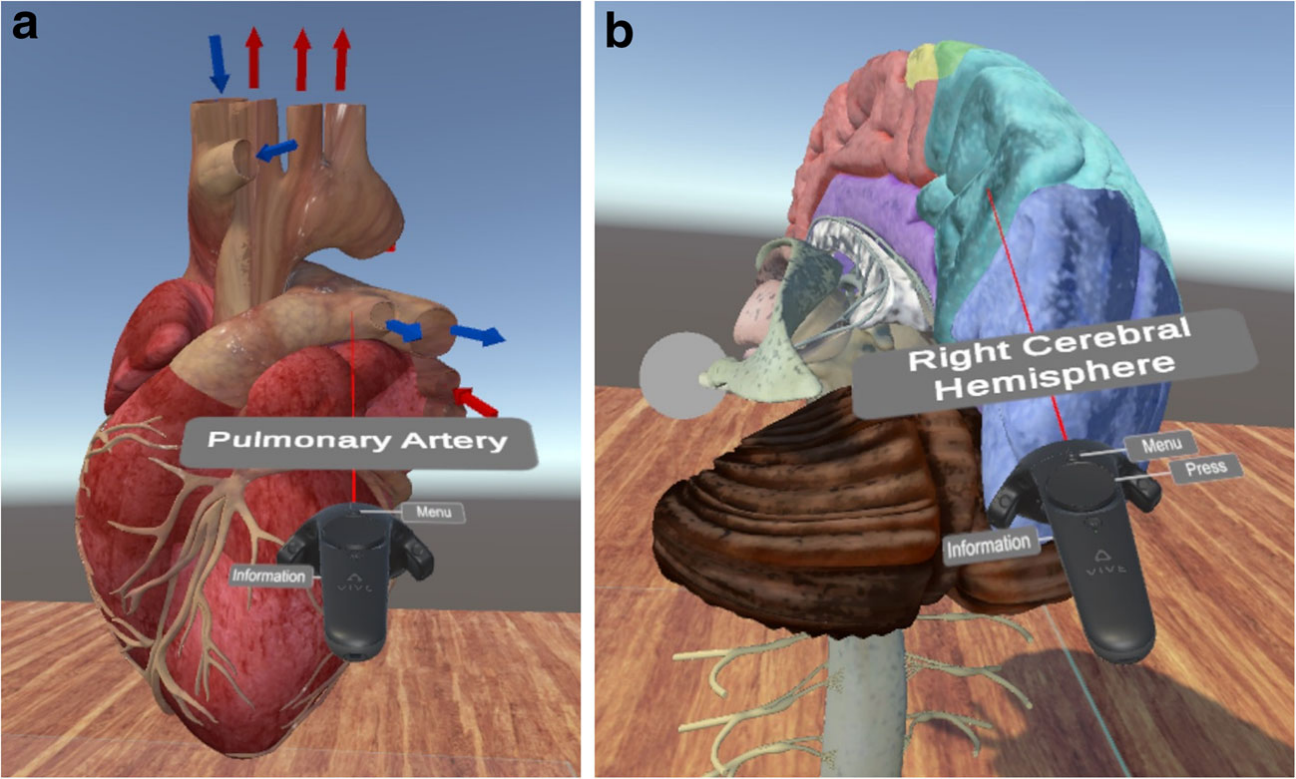
Example of two labeled models: **a** Heart. **b** Brain

VR4Health is designed to be used in an HMD device. This implies a necessary isolation of the user because he/she is completely immersed into the VR world and does not have any contact to the real world around him/her. The application is directed to an individual use in order to be a self-learning tool that the student can use everywhere. In this sense, the different virtual models shown in VR4Health are completely labeled with all parts and sub-parts easily identifiable by the student (see Fig. 1). These labels were decided taking into account the student necessities identified by teachers. The VR application has also visual and sound feedback in order that the user knows that he is interacting with it. It also includes multi-language capacity (at the moment 3 languages available: Catalan, Spanish and English) that allows to easily configure any other language by only writing the correct names into a configuring file.

The application starts with a virtual world that visualizes, onto a 3D wall, a menu showing all the possible anatomical structures to visit. In this step, the user can select which structure to visit and the virtual world changes to become the chosen anatomical structure which is scaled to be big enough to be visited and inspected in detail by the user. For this inspection we use *VR implicit interaction* by recognizing the movements of the users in the real world and making them be the same movements into the virtual world. The application also allows the user to cut models through a plane in order to see slices of the model.

Apart from the labeling included in all models, each one of them has been also improved with some other particular visual information. In Table 1 we describe these characteristics for each one.

**Table 1:** Description of models and their animations

*Heart*	Colored arrows indicating the direction and kind of blood. Animated
(by *3dregenerator*)	blue or red *pills* flowing through it. Vertical section view.
*Eye* (by *Alef itd*)	Animation separating the layers showing all the different parts.
*Ear* (by *Imagework*)	Colored different sections. Animation simulating sound into the ear.
*Circulatory system*	Visualization of arteries and veins. Semitransparent view of skin and
(by *dugongmodels*)	bones.
*Digestive system*	Adding liver, pancreas and gallbladder. Animation showing the
(by *3d moliere and Activepoly*)	exocrine and endocrine secretion and the pancreatic circulation.
*Lungs*	Animation and a zoomed vision of an alveol. Colored blue and red
(by *scyrus*)	arrows simulating blood. Animation simulating the air into the lungs.
*Brain*	Possible division in colored parts. Show separately ventricular system,
(by *leo3Dmodels*)	basal ganglia and nervous system.

The application includes at the moment these seven 3D anatomical models but it is completely scalable so it allows to add any other anatomical model or substitute one of them. Teachers can suggest any modification of models or animations although they need to be done by a technician.

### Teacher helping mechanisms

The student can inspect all the structures he/she wants and at the same time the application is registering what the student is doing during his/her inspection in order to help the teacher to facilitate the learning flow.

The information the application registers is: *What structures the student inspected*, *How long the student dedicated to each structure* and *How many times each label into each structure has been required*.

With all this information captured by the application during the students inspection, the teacher can evaluate different aspects about the student’s learning:

- *What structures inspected:* With this information the teacher knows what the student is visualizing and can direct him/her to a more guided session if needed. This information is also useful to know at any time what each student has worked through and the teacher can ask about it.
- *Time dedicated to each structure:* This gives information about the time each student devotes to each structure and whether there is any structure that has not been seen or has not been seen with enough time. The time devoted to the learning of each structure is important for the teacher because it encourages him to ask himself why and to ask the student the reason (greater difficulty in learning, greater interest...).
- *How many times each label has been required:* The option knowing how many times the student needed to see a label with the name of the structure of substructure gives to the teacher idea of the previous knowledge of the student. The excess of consultancy or the never consultancy of a label can alert to the teacher and push him/her to ask to the student for the reason (no motivation, lack of study, ...).

All this information stored by the application can give also an overall view to the teacher in order to know whether the student needs a bit more help on this learning or not, or if there is any structure shown in the application that should be better explained in some sense.

## Experiment design

The experiment was designed to analyze the goodness and/or difficulties found by the students in the use of the VR4Health project for the autonomous learning and the advantages teachers can have by using the information that the application gets from the student’s participation.

### Sample and participants

The participants in the experiment was established with 6 teachers of human anatomy and 18 students enrolled in subjects of anatomy and human physiology. The students were from two different degrees in health sciences, both taught at the same institution and where the contents of human anatomy are contemplated in both cases in the first year of training.

The inclusion criteria of students was to be enrolled in the subject of human anatomy. As criteria of exclusion, those students repeating the subject or having completed an education with human anatomy contents.

The inclusion criteria of teachers was to teach human anatomy at the Campus Docent Sant Joan de Déu.

The experiment was done between December 2018 and February 2019. First contact with the participants was made in December. The rest of the participants were achieved through the snowball strategy. Once the researcher came into contact with the future participant, he checked the eligibility to participate and the information and confidentiality document was delivered to him. With the help of a technician the test was conducted individually and had a maximum duration of one hour.

During the test no feedback was given to the participant in order not to condition the action of the participant. The participant had total freedom of decision over which models to inspect, what to visualize in them and how much time to devote to them based on their interests, concerns of knowledge or exploration.

At the end of the test a questionnaire was delivered to the participant and we previously explained the purpose and length of the questionnaire as well as the authorship.

### Ethical considerations

Given the nature of the study, it was not necessary to approve the ethics committee. Information document and informed consent were signed before testing the application. Participants were reinformed on anonymity and confidentiality as well as warned that no personal data would be collected, the data was stored in a safe place. Participants were informed about the benefits and possible damages and that the withdrawal of the project would not affect their attention, relationship or their grades in the case of students.

### Validity and reliability

The written questionnaire was specifically developed for the presented experiment by the researchers, taking into account the previous knowledge, skills and abilities of the students. Once the draft questionnaire was finished, it was reviewed by a nurse with experience in the design of learning spaces and the nursing curriculum and two technologists, an expert in programming VR applications and systems and the other expert in research in VR.

To obtain consistency of the questionnaire it was reviewed by 3 students of the first year, with 4 questions that were modified following the suggestions of the students.

## Evaluation and results

The evaluation is composed by two different parts:

- *The questionnaire*, which includes data on 3 categories: *(1) Ease of use*, *(2) Self-learning usage* and *(3) Teacher helping usage*. The questionnaire had 8 closed questions in which the student had to indicate the degree of agreement or disagreement with a score of 1 to 6 (1 = completely disagree, 6 completely agree). It has also 7 open questions in order to value the preferences on the teacher helping (category 3).
- *Application data*, which includes data on measure the time each user spent in any structure and data on how many times the substructure label has been visited or required. This information is captured automatically by the application for the teacher helping mechanism (see Section “Teacher helping mechanisms”) and it is also giving us results about which structures are more visited by the users.

### Questionnaire

Questions asked to the user organized in the 3 categories are presented in Tables 2 and 3.

**Table 2:** Questions for categories 1 and 2

Category 1. Ease of use:	
Q1.1	The use of a Head Mounted Display (HMD) has been comfortable?
Q1.2	The use of the VIVE hand controller has been easy?
Q1.3	The navigation through the structures menu has been intuitive?
Category 2. Self-learning usage:	
Q2.1	By using VR, do you understand better the anatomical structures?
Q2.2	By using VR, do you understand better the relative position of the different structures in the body?
Q2.3	By using VR, have you been more motivated than using traditional study?
Q2.4	The anatomical models you have inspect in VR4Health are a good support material for the human anatomy course?
Q2.5	Would you use VR4Health as a support material for your classes?

**Table 3:** Questions for category 3

Category 3. Teacher helping usage:	
Q3.1 - How do you prefer to use VR4Health? (2 max. answers ordered)	
1)	Individually with teacher support online
2)	Individually without teacher support online
3)	In group of students with teacher support online
4)	In group of students without teacher support online
5)	Individually with teacher support presentially
6)	In group of students with teacher support presentially
Q3.2 - With respect to the time of use, do you prefer an open time (no limit) or having a time limit proposed by the teacher? Why?	
1)	Without limits
2)	With limits
3)	Both options
Q3.3 - Do you prefer a predefined and guided inspection through the anatomical structure or do you prefer the student freely deciding it?	
1)	Defined
2)	Free
3)	Mixed
Q3.4 - Do you find useful that the teacher receive information about the inspection the student has done through the anatomical structures	
(what structures visited, how long for each one, etc...) in order to know the learning necessities of the student?	
Q3.5 - Do you find useful that teachers give feedback to the student from the data analysis of the information obtained through the application?	
Q3.6 - How do you think this feedback should be given to the student? (2 max. answers ordered)	
1)	Through e-mail
2)	Through the application
3)	Through moodle or a similar teaching application
4)	Presentially
5)	Through Skype or facetime
6)	Individually
7)	Collectively
Q3.7 - When do you think feedback should be done? (2 max. answers ordered)	
1)	During the use of the application
2)	Immediately after using the application
3)	During the same day of using the application (24h)
4)	During the week of using the application
5)	While implementing the course (semester)
6)	Other:

### Results from questionnaire

1. Ease of use:
  The answers to the 3 questions in this category are all in the range [4:6] so Table 4 are only showing those 3 values.

**Table 4:** Results of questions in Category *(1)* (Q1.1, Q1.2 and Q1.3)

Q1.1	Students	Teachers	Q1.2	Students	Teachers	Q1.3	Students	Teachers
4	1 (5.6%)		4	1 (5.6%)	1 (16.7%)	4		
5	9 (50%)	3 (50%)	5	4 (22.2%)		5	6 (33.3%)	3 (50%)
6	8 (44.4%)	3 (50%)	6	13 (72.2%)	5 (83.3%)	6	12 (66.7%)	3 (50%)
  From the answers we can say the users felt comfortables with the VR system proposed and also with the user interface that they found very intuitive.
2. Self-learning usage:
  In this category we can say that the general opinion of students and teachers is that the use of VR helps on the understanding of the 3D shapes of the anatomical structures and also on the relative position each one has into the human body (see Table 5).

**Table 5:** Results of questions in Category *(2)*

Q2.1	Students	Teachers	Q2.2	Students	Teachers	Q2.3	Students	Teachers
4	2 (11.1%)		4			4	1 (5.6%)	
5	1 (5.6%)		5	6 (33.3%)	1 (16.7%)	5	3 (16.7%)	1 (16.7%)
6	15 (83.3%)	6 (100%)	6	12 (66.7%)	5 (83.3%)	6	14 (77.8%)	5 (83.3%)
Q2.4	Students	Teachers	Q2.5	Students	Teachers			
4			4					
5	2 (11.1%)		5	3 (16.7%)				
6	16 (88.9%)	6 (100%)	6	15 (83.3%)	6 (100%)
  The users also consider that using VR is more motivating and they would use the application as a support material for the human anatomy course.
3. Teacher helping usage:
  In the third category we have grouped the 7 questions in 3 groups: *How to use the application*, *Obtaining feedback* and *How and when to obtain feedback*.
  How to use the application:
    From the answers given to questions Q3.1, Q3.2 and Q3.3 (Fig. 2) we can say the students prefer to use the application individually and with the teacher present in order to obtain support presentially. Teachers, however, prefer group sessions but giving either presentially or on-line support.

**Fig. 2:**
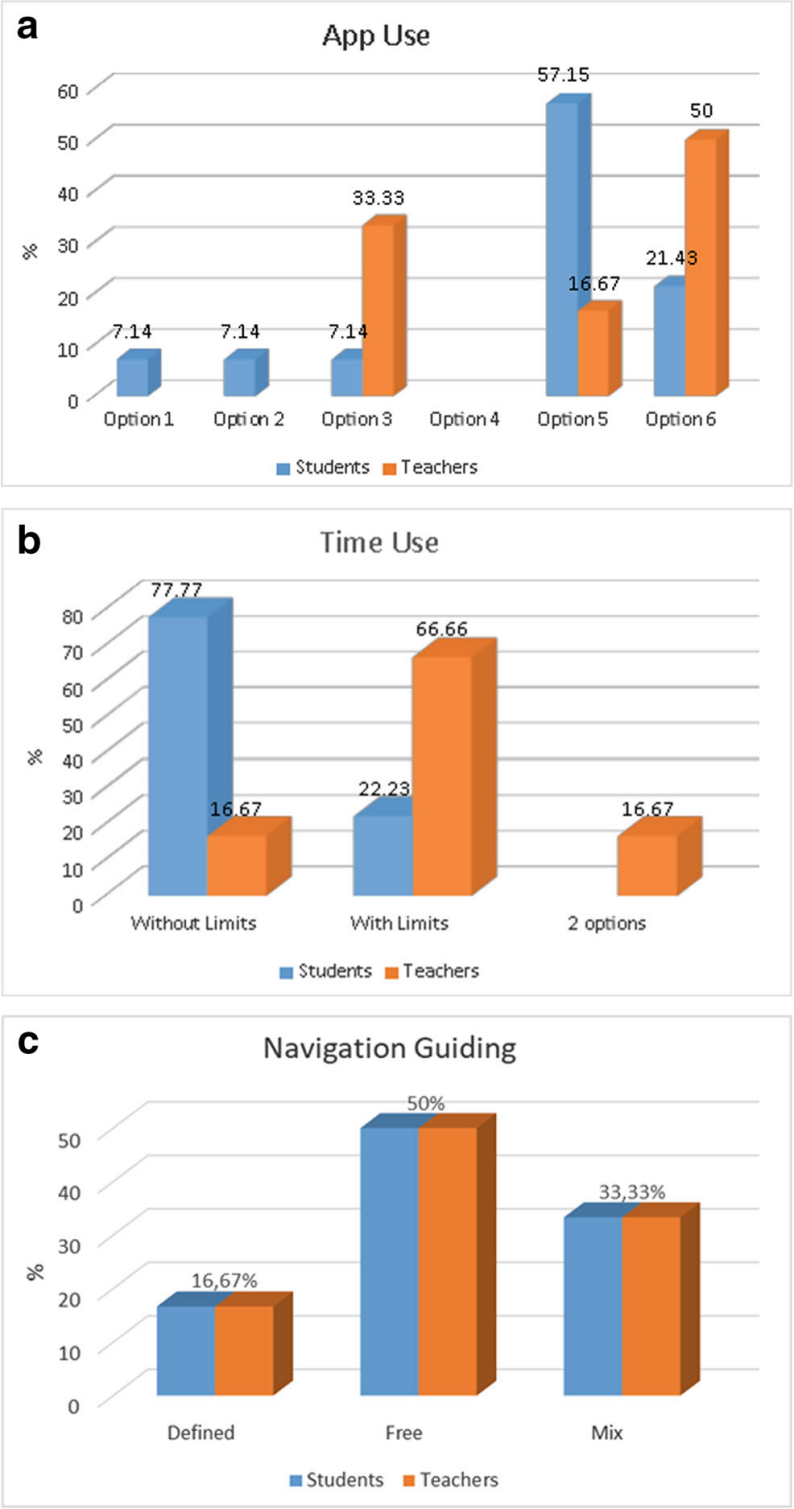
Answers to questions: **a** Q3.1. **b** Q3.2. **c** Q3.3
    Students also prefer to have unlimited time to use the application, while teachers prefer to force a limited time of usage.
    Both students and teachers prefer to use the application freely instead of a guided use or, at maximum, be guided only in a part of the usage.
  Obtaining feedback:
    Both, students and teachers, agree on the importance of getting information from the application in order that the teacher can give feedback to the student and help him/her with the material inspected (see Table 6).

**Table 6:** Results of questions *Q3.4* and *Q3.5*

Q3.4	Students	Teachers	Q3.5	Students	Teachers
Yes	16 (88.9%)	6 (100%)	Yes	16 (88.9%)	6 (100%)
No	2 (11.1%)		No	2 (11.1%)
  How and when to obtain feedback:
    With respect to the question of how to obtain this feedback (Q3.6), both students and teachers consider it should be given presentially and individually (see Fig. 3a). Surprisingly, some teachers also prefer the possibility of giving feedback through the application or other teaching application (like moodle) but these are not options preferred by the students (only one student on each option 1, 2 or 3).

**Fig. 3:**
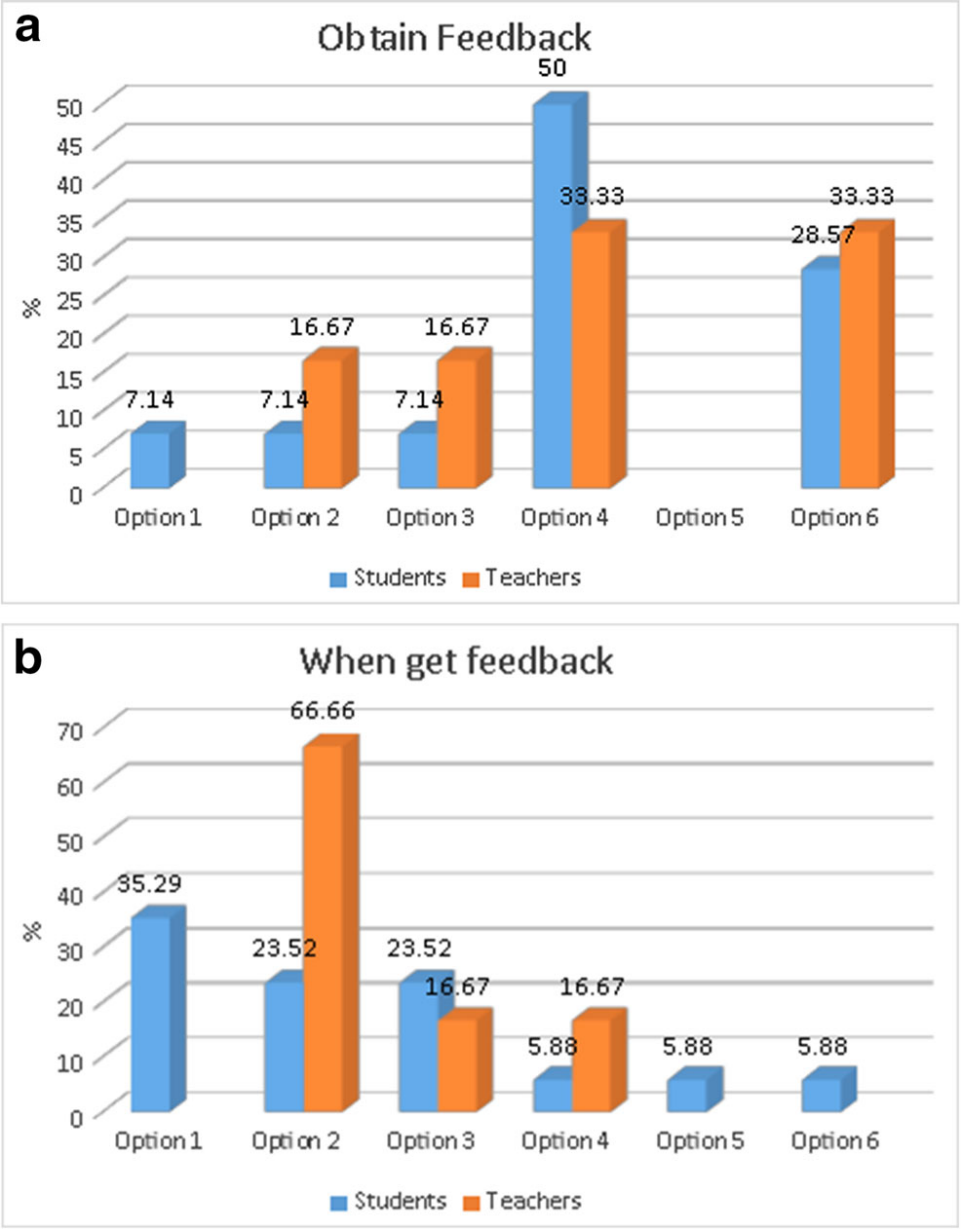
Answers to questions: **a** Q3.6. **b** Q3.7
    Students prefer to get the feedback during the use of the application (Q3.7 in Fig. 3b) while teachers clearly opt to give it immediately after the use of the application and none of them choose to do it during the use of it (option 1).

### Results from application data (usage results)

**Fig. 4:**
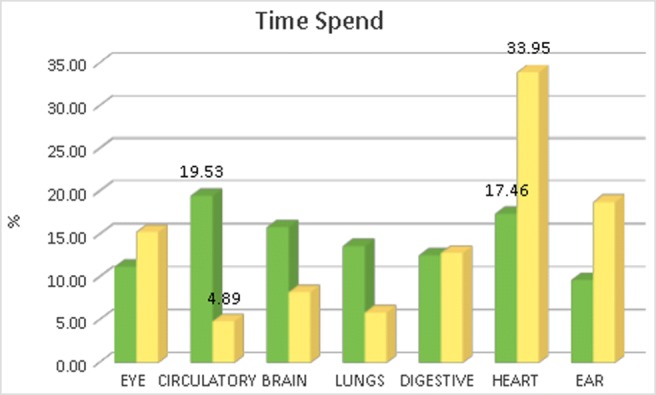
Time spent by the user in each structure. Green bars: total time spent in the structure. Yellow bars: time with respect to the number of substructures

Following the information obtained for the teacher helping usage we can also study the time each user has spent in any of the anatomical structures available in the application (see Fig. 4).

From this data we can see that taking into account the total time spent in a structure (green bars in Fig. 4) the *Circulatory system* has been the most inspected. But this total time is not fair because the number of labeled substructures existing in any anatomical structure is different, so we should take into account the time spent with respect to the amount of substructures existing in the anatomical organ (see Table 7). Taking this measure into account (yellow bars in the figure) we can observe the *Heart* is the most inspected organ so we can say the most interesting for the users (both, students and teachers).

**Table 7:** Number of labels existing in each anatomical structure

Structure	Tag names
Eye	20
Circulatory	112
Brain	59
Lungs	63
Digestive	30
Heart	14
Ear	16

By being asked about what part of the application they found more interesting, users said they appreciate a lot the animations offered into the different anatomical structures, like blood flowing inside the heart, air coming into the lungs or the movement of the different parts in the ear system when it simulates sound coming in.

## Conclusions

The results presented show that for students and teachers VR4Health is a self-learning tool that facilitates the understanding regarding the volume and the relationship among the different anatomical structures. However they have some differences regarding on how to use the application.

A first difference is that being a tool designed to favor self-learning, students prefer the presence of the teacher to obtain support in the learning process and in a face-to-face way in front to other options (virtuality, mailing, teleconferencing, etc.). This suggests that students prefer a mixed training program and a tool that promotes blended-learning instead of self-learning. This result coincides with the study of ECAR [8].

A second difference is that students prefer to use the application individually while the teachers prefer it in a group manner. This is attributed to the students desire of autonomy and centrality in the learning while the professors prioritize the collaborative learning by the benefits of sharing knowledge and experience, although respecting the individuality and the centrality at the time of providing feedback. In spite of the divergence of preferences, the tool responds to the wishes of both groups at once, giving full student leadership in their learning and needs, and providing individual student information to the teacher so that he can build the feedback and ask the student about his commitment and behavior during the training time.

The last difference encountered refers to the moment of giving feedback, students prefer to have feedback already during the use of the application while teachers prefer to do it immediately at the end of the student’s session. Since the application is directed to self-learning the requirement coming from the students is not possible. In case of other learning methodologies the students appreciations should be taken into account.

The experiment has also shown that technological development in VR allowed the design and development of a tool that fits in the learning paradigms promoted to the present (self-learning and blended learning), but it must be pointed out the existence of certain limitations that may affect the results. The first is that not all participants took the pilot test on the same day. The existence of an anatomy exam next to one of the days of testing may have introduced modifications regarding the time of visualization of certain models and the access to some labels by the participants of that day. The second is that the students participating in the experiment come from two different degrees but no results have been compared and the different methodology used in each degree to teach and learn anatomy may have conditioned the student’s motivation during the use of the application.

We can conclude that VR4Health is a tool that is perceived by users as an easy-to-use tool for self-learning of human anatomy and physiology and that covers the need for learning support manifested so for students as well as for teachers. In addition, the tool facilitates the change in the role of the teacher who goes from being an anatomy teacher (expert in the field of anatomy) to a facilitator of learning (expert in the learning of anatomy), a change of role that responds to the need to level the student-teacher relationship and return the protagonism of the learning process to the student who has to assume responsibility for their own learning.

As a future work, once we know the students and teachers perception about the application, we can think in some improvements offered by new technology, specifically in two aspects:

- *Direct interaction with 3D models*: We plan to add to the application some touching interaction by using a haptic device (for example glove) in order to simulate organ deformations by touching them.
- *Improving engagement*: We can add some gamification strategies like rankings, marks, etc. and also collaborative Virtual Reality techniques in order to allow the interaction with other users into the application. Both approaches would improve the student engagement.
